# Cohort Profile: Norwegian Offshore Petroleum Workers (NOPW) Cohort

**DOI:** 10.1093/ije/dyaa107

**Published:** 2020-09-02

**Authors:** Jo S Stenehjem, Ronnie Babigumira, H Dean Hosgood, Marit B Veierød, Sven Ove Samuelsen, Magne Bråtveit, Jorunn Kirkeleit, Nathaniel Rothman, Qing Lan, Debra T Silverman, Melissa C Friesen, Trude E Robsahm, Kristina Kjærheim, Bettina K Andreassen, Nita K Shala, Fei-Chih Liu, Leif-Åge Strand, Tom K Grimsrud

**Affiliations:** 1 Department of Biostatistics, Oslo Centre for Biostatistics and Epidemiology, University of Oslo, Oslo, Norway; 2 Department of Research, Cancer Registry of Norway, Oslo, Norway; 3 Division of Emergencies and Critical Care, Oslo University Hospital, Norway; 4 Department of Epidemiology and Population Health, Albert Einstein College of Medicine, The Bronx, NY, USA; 5 Department of Mathematics, University of Oslo, Oslo, Norway; 6 Department of Global Public Health and Primary Care, University of Bergen, Bergen, Norway; 7 Department of Occupational Medicine, Haukeland University Hospital, Bergen, Norway; 8 Division of Cancer Epidemiology and Genetics, Occupational and Environmental Epidemiology Branch, National Cancer Institute, Bethesda, MD, USA; 9 Institute of Military Medicine and Epidemiology, Norwegian Armed Forces Joint Medical Services, Sessvollmoen, Norway

## Why was the cohort set up?

The NorwegianOffshore Petroleum Workers (NOPW) cohort is a cohort that recruited nearly 28 000 offshore workers in 1998 for prospective follow-up of cancer and cause-specific mortality. The cohort was based on a list of possible former or current offshore workers who were invited to fill in and return a comprehensive questionnaire on work history, diet, alcohol, tobacco, education and other factors possibly related to cancer risk (Supplemental material, available as Supplementary data at *IJE* online).

In 1963, Norway proclaimed sovereignty of the continental shelf along the Norwegian coastline and its natural resources.^1^ From 1966, exploration and drilling for oil and gas in the North Sea eventually resulted in a large number of wells that were operated from movable and stationary installations, with production starting in 1971. In this pioneer time of the industry, there were few automated processes and the work was highly manual, physically demanding and dirty, with relatively high injury and death rates from accidents.^2^^,^^3^ Health and safety regulations were scarce and the use of personal protective equipment limited.^1^^,^^4^ The weather conditions offshore are harsh, and the workers are subject to a wide range of exposures: chemical, physical, ergonomic and psychosocial.^5^ Some of the chemical exposures are known or suspected to be carcinogenic, and exposure to natural gas and chemicals from water injection, oil/solvent vapour, exhaust fumes and skin contact with oil and diesel have been frequently reported by the workers.^5^

The first questions about cancer risk related to drilling and production of oil and gas on the Norwegian continental shelf were raised in the early 1980s. It was pointed out that the possible carcinogenic effects from long-term low-level hydrocarbon exposure and other chemical agents required continuous monitoring due to long latency time.^6^^,^^7^ In 1990, a review of health effects from exposure to oil-based drilling fluids concluded that there is insufficient information of such exposure, especially regarding carcinogenicity and pathological changes in the lungs.^8^ The living conditions and working environment offshore led the oil and gas employers’ association, labour unions and the Cancer Registry of Norway (CRN) to plan a follow-upstudy of cancer incidence and cause-specific mortality among Norwegian offshore workers. In 1992 the CRN issued a research protocol,^9^ but it proved impossible to establish a uniform and complete historical cohort of offshore petroleum workers—neither by means of data from employers nor from census and registry data. Because of this situation, the CRN planned and conducted the above-mentioned recruitment of participants for the prospective cohort study by compiling lists of possible offshore workers from oil companies, educational institutions, unions and other relevant sources.^10^

## Who is in the cohort?

Among 57 329 workers with possible employment in the offshore petroleum industry, 35 458 (62%) returned the questionnaire (Supplementary Table S1, available as Supplementary data at *IJE* online). After excluding those who reported no offshore work (*n* = 7249), had missing address (*n* = 222), worked on ships with no drilling or production activity (*n* = 68) or had missing personal identification number (PIN: *n* = 2), the NOPW cohort consisted of 27 917 workers (79% of all responders). Since the true number of all offshore workers is unknown for the period 1965–98, we merged the NOPW cohort with the Norwegian State Register of Employers and Employees (NREE) based on first entry in offshore work after 1980 (although limited to data on type of work and duration) according to a protocol for another registry-based cohort study of Norwegian offshore petroleum workers.^11^ From these two independent sources, we estimated the participation rate in the NOPW cohort for the period 1981–98 to be 69%.^12^

Table 1 shows age, sex and county of residence for the NOPW cohort members and the non-responders at the time of recruitment in 1998. Age was similar in cohort members and non-responders (43.1 vs 42.8 years, respectively). Male workers constituted a slightly larger proportion among the cohort members (90.8%) than among the non-responders (88.6%). Differences in county of residence were small; 23.1% of the cohort members vs 19.7% of the non-responders resided in Hordaland, and correspondingly 31.4% vs 36.3% in Rogaland, which constituted the two most relavent counties.

**Table 1.** Age, sex and county of residence for participants in the Norwegian Offshore Petroleum Workers (NOPW) cohort and non-responders at baseline in 1998

	**NOPW cohort (*n* = 27 917)** ^a^	**Non-responders (*n = *21 871)** ^a^
Age in 1998 (years), mean (SD)	42.6 (9.8)	42.8 (10.2)
Sex, *n* (%)		
Males	25 347 (90.8)	19 381 (88.6)
Females	2570 (9.2)	2490 (11.4)
County in 1998, *n* (%)		
Akershus	710 (2.5)	667 (3.0)
Aust-Agder	1302 (4.7)	1032 (4.7)
Buskerud	500 (1.8)	411 (1.9)
Finnmark	70 (0.3)	67 (0.3)
Hedmark	152 (0.5)	114 (0.5)
Hordaland	6440 (23.1)	4301 (19.7)
Møre og Romsdal	1249 (4.5)	802 (3.7)
Nord-Trøndelag	500 (1.8)	297 (1.4)
Nordland	491 (1.8)	316 (1.4)
Oppland	154 (0.6)	100 (0.5)
Oslo	622 (2.2)	793 (3.6)
Rogaland	8769 (31.4)	7945 (36.3)
Sogn og Fjordane	700 (2.5)	363 (1.7)
Sør-Trøndelag	842 (3.0)	620 (2.8)
Telemark	1107 (4.0)	715 (3.3)
Troms	311 (1.1)	224 (1.0)
Vest-Agder	1837 (6.6)	1415 (6.5)
Vestfold	1552 (5.6)	1240 (5.7)
Østfold	606 (2.2)	449 (2.1)

SD, standard deviation.

a Missing county on three NOPW cohort members (*n* = 27 914) and four non-responders (*n* = 21 867).

**Figure 1:**
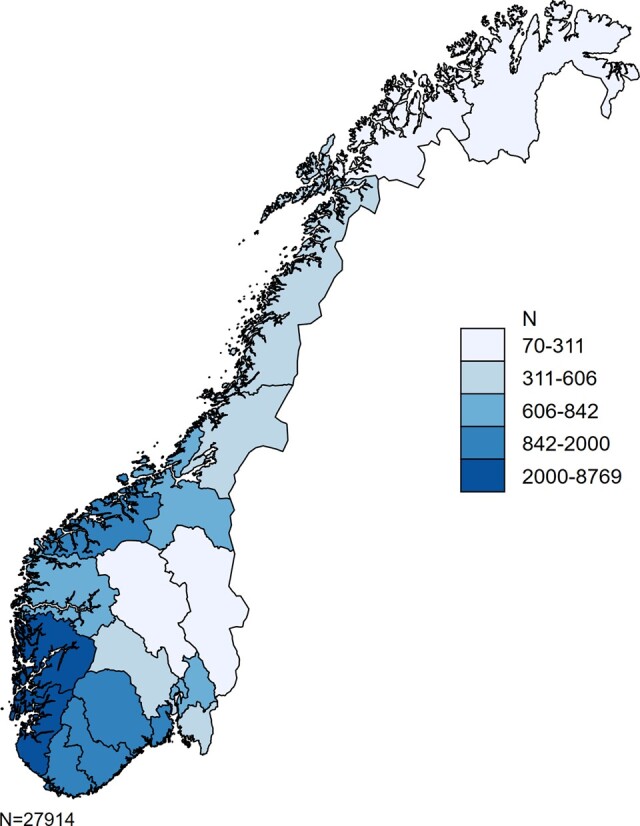
Map of Norway showing the county of residence for the members of Norwegian Offshore Petroleum Worker (NOPW) cohort at the baseline (1998), displayed as number of workers on a five-category colour scale (*n* = 27 914; missing county on three workers).

**Figure 2:**
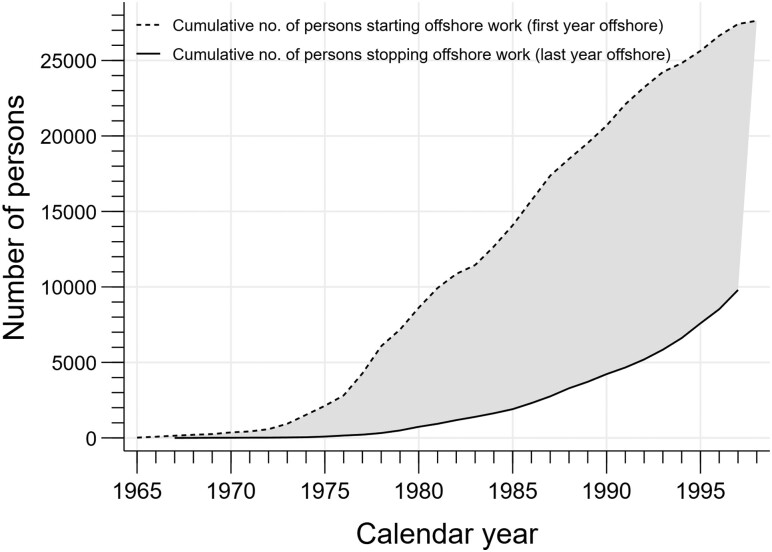
Cumulative number of persons starting (dotted line) and stopping (solid line) offshore work in the NOPW cohort over the time period 1965–98.

All counties of Norway are represented in the NOPW cohort, where workers residing in the northernmost and inland counties constituted the smallest fractions (Figure 1). The cumulative numbers of persons starting (dotted line) and stopping (solid line) offshore work over the time period 1965–98 are shown in Figure 2. A steep increase in the number starting offshore work was seen during the 1970s and 1980s.

Start of cancer follow-up was set to 1 July 1999 to allow for delayed questionnaires to be received before starting the follow-up time, and thereby ensuring a prospective follow-up through 31 December 2017. The linkage was conducted by use of the unique 11-digit PIN assigned to all Norwegians in 1964 for those alive in 1960 or born later. Reporting of incident cancers to the CRN has been compulsory in Norway since 1953, and data from a number of sources ensure a high degree of completeness and validity.^13^ A total of 3868 and 303 cases were identified among 25 347 male and 2570 female workers, respectively (Table 2). Among cancer sites possibly related to occupational exposure, we computed standardized incidence ratios (SIRs) with 95% confidence intervals (CIs) from gender-, age (5-year)- and time (1-year)-specific incidence rates for the Norwegian population (Table 2). We assumed a Poisson distribution of the observed cases. Analyses were performed using Stata 16.1 (StataCorp, TX, USA). A total of 568 prevalent cancer cases (i.e. occurring 1955–30 June 1999) were excluded.

**Table 2.** Standardized incidence ratios (SIRs) with 95% confidence intervals (CIs), by sex, in the Norwegian Offshore Petroleum Workers (NOPW) cohort (*n* = 27 917), 1999–2017

Cancer site	ICD-10	Males (n = 25 347)			Females (n = 2570)		
		Obs.	Exp.	SIR (95% CI)	Obs.	Exp.	SIR (95% CI)
Oral cavity and pharynx	C01–C14	73	80.7	0.90 (0.71–1.14)	2	3.0	0.67 (0.08–2.43)
Oesophagus	C15	41	43.2	0.95 (0.68–1.29)	2	0.9	2.26 (0.27–8.16)
Adenocarcinoma	C15	27	24.2	1.12 (0.74–1.63)	1	0.3	3.45 (0.09–19)
Squamous cell carcinoma	C15	12	13.7	0.87 (0.45–1.53)	1	0.5	2.12 (0.05–12)
Colorectal	C18–C21	431	446.0	0.97 (0.88–1.06)	33	28.0	1.18 (0.81–1.66)
Larynx	C32	24	26.8	0.89 (0.57–1.33)	1	0.4	2.56 (0.06–14)
Lung	C34	386	356.6	1.08 (0.98–1.20)	23	21.1	1.09 (0.69–1.64)
Small-cell lung cancer	C34	53	54.8	0.97 (0.72–1.26)	5	3.9	1.29 (0.42–3.01)
Non-small-cell lung cancer	C34	333	301.8	1.10 (0.99–1.23)	18	17.2	1.05 (0.62–1.66)
Pleura	C38.4	32	13.5	2.38 (1.63–3.36)	0	0.1	—
Cutaneous melanoma	C43	214	219.4	0.98 (0.85–1.12)	32	19.8	1.62 (1.11–2.29)
Cutaneous squamous cell carcinoma	C44	133	119.3	1.12 (0.93–1.32)	10	6.1	1.65 (0.79–3.04)
Breast	C50	12	5.5	2.18 (1.13–3.81)	99	86.3	1.15 (0.93–1.40)
Prostate	C61	1277	1060.1	1.20 (1.14–1.27)			—
Kidney	C64	136	130.4	1.04 (0.87–1.23)	4	4.4	0.91 (0.25–2.33)
Bladder	C66–C68	213	214.2	0.99 (0.87–1.14)	6	5.1	1.18 (0.43–2.58)
Lymphohaematopoietic	C81-C96, D45-D47	292	312.3	0.93 (0.83–1.05)	16	17.4	0.92 (0.52–1.49)
Hodgkin lymphoma	C81	12	15.5	0.77 (0.40–1.35)	0	0.9	—
Non-Hodgkin lymphoma (NHL)	C82–C91	206	225.1	0.91 (0.79–1.05)	11	11.9	0.92 (0.46–1.65)
Follicular lymphoma	C82	29	28.1	1.03 (0.69–1.48)	2	2.2	0.93 (0.11–3.35)
Mantle cell lymphoma	C83.1	13	8.9	1.46 (0.78–2.50)	0	0.2	—
Diffuse large B cell lymphoma	C83.3	45	36.2	1.24 (0.91–1.66)	2	1.8	1.10 (0.13–3.96)
Multiple myeloma	C90	48	47.8	1.00 (0.74–1.33)	1	2.5	0.40 (0.01–2.24)
Acute lymphoid leukaemia	C91.0	5	2.5	1.97 (0.64–4.61)	0	0.2	—
Chronic/small lymphoid leukaemia	C91.1	35	39.0	0.90 (0.63–1.25)	2	1.6	1.24 (0.15–4.48)
Acute myeloid leukaemia	C92.0	25	20.9	1.20 (0.77–1.77)	5	1.3	3.76 (1.22–8.78)
Chronic myeloid leukaemia	C92.1	8	6.0	1.33 (0.57–2.61)	0	0.4	—
Myelodysplastic syndrome	D46	17	18.8	0.90 (0.53–1.45)	0	0.8	—
All sites	C00-C96, D45-D47	3868	3602.0	1.07 (1.04–1.11)	303	268.3	1.13 (1.01–1.26)

ICD, International Classification Of Diseases; Obs., observed; Exp., expected.

**Figure 3:**
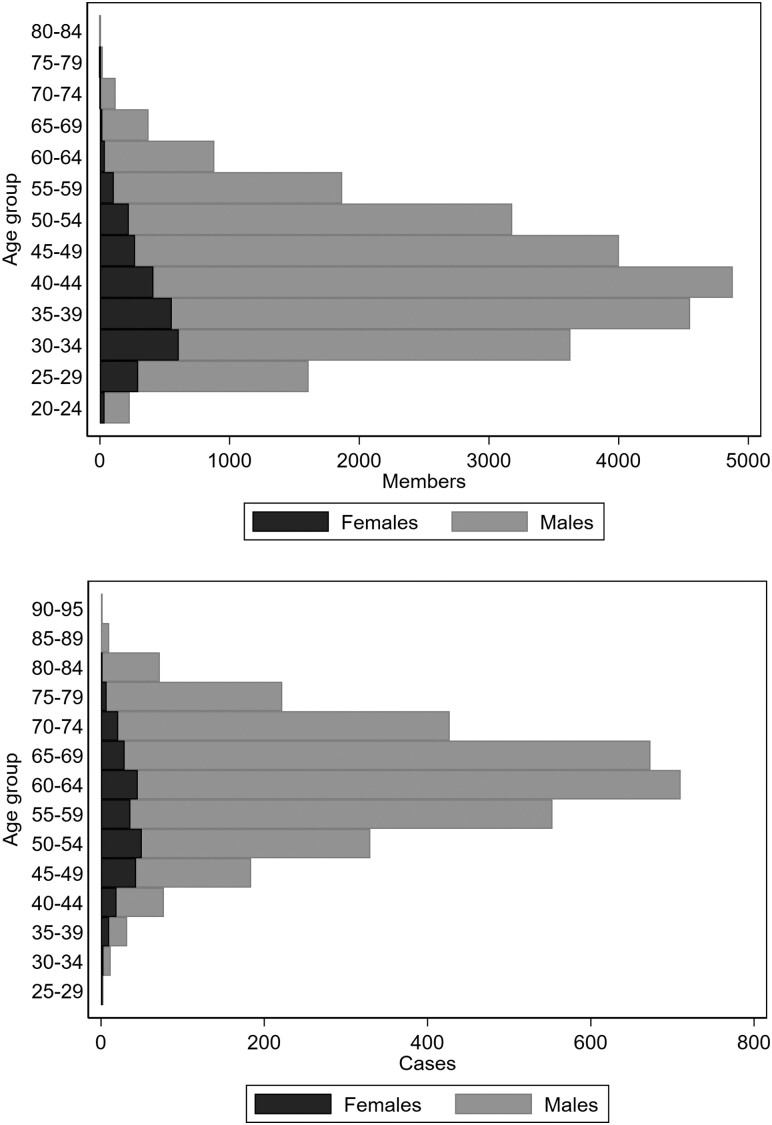
Age distribution by sex among cohort members at baseline in 1998 (upper panel) and among cancer cases at diagnosis 1999–2017 (lower panel).

Figure 3 shows age distribution by sex among all cohort members at baseline (upper panel) and among the incident cancer cases at diagnosis (lower panel). The largest 5-year age group at baseline was 40–44 among males and 35–39 among females. Most male cancer cases occurred after age 59, whereas for females age groups were more evenly distributed, primarily because most breast cancer cases were diagnosed before age 54.

## How often have they been followed up?

The cohort has been linked to the CRN five times thus far. The first linkage was conducted with follow-up through 2005 and yielded 773 incident cases.^2^^,^^14^ The second follow-up was through 2009 and yielded 1585 incident cases.^12^ The third and fourth follow-ups were conducted through 2011^15^ and 2012,^16^^,^^17^ respectively. The fifth and current follow-up runs through 2017 and has yielded 4171 incident cases.

Necessary legal and ethical approvals were obtained from the Norwegian Data Inspectorate, the Regional Committee for Medical Research Ethics and the Norwegian Directorate of Health.

## What has been measured?

The questionnaire at baseline was comprehensive, covering sociodemographic factors, work history before, after and in-between offshore work periods, and lifestyle factors.

Table 3 shows cohort characteristics relevant for cancer risk. More than 70% of the workers were born after 1950. Males were slightly older than females at recruitment (mean ages 42.9 and 39.1 years, respectively). A larger proportion of males (79%) had a partner than females (66%), but a lower proportion of males (18%) were childless than females (31%). Females with children were aged on average 25 years at first childbirth. Vocational training was the most frequent educational category among males (41%) and upper secondary education among females (33%). Among males, 55% were overweight or obese (body mass index ≥25 kg/m^2^), as were 25% of the females. The average number of offshore jobs was around two among males and 1.5 among females, and corresponding means of total offshore employment duration were 11 and 7 years, respectively. Maintenance activities constituted the largest work category among males (50%), whereas most females were engaged in catering, office and administrative work (68%); 41% of the males and 35% of the females reported shift work in their last position. Nearly 70% of the workers were either former or current smokers, with an average smoking history of 11.4 pack-years. Alcohol and red meat intake was divided into quartiles, and quartile 4 corresponded to ≥4.1 alcohol units/week (males 23%, females 8%) and red meat ≥25.1 times/week (males 24%, females11%). Physical activity with an intensity at the aerobic threshold (sweaty and short of breath) for ≥20 min was performed 1–2 times/week by 26% of the males and 30% of the females. During a year, 63% reported one sunburn and 2–3 weeks of sunbathing; 7% of the males and 16% of the females reported using a solarium 1–2 times a month, and 26% of the males and half the females reported using sunscreen almost always.

**Table 3.** Baseline characteristics of the participants in the Norwegian Offshore Petroleum Workers (NOPW) cohort

Variables	Males (n = 25 347)	Females (n = 2570)	Total (n = 27 917)
***Socio-demographic***			
5-year birth cohorts, *n* (%)			
1918–29	190 (<1)	7 (<1)	197 (<1)
1930–34	461 (2)	22 (1)	483 (2)
1935–39	1017 (4)	50 (2)	1067 (4)
1940–44	2173 (9)	134 (5)	2307 (8)
1945–49	3355 (13)	222(9)	3577 (13)
1950–54	4204 (17)	300 (12)	4504 (16)
1955–59	4931 (19)	441 (17)	5372 (19)
1960–64	4361 (17)	594 (23)	4955 (18)
1965–69	3309 (13)	534 (21)	3843 (14)
1970–74	1211 (5)	243 (9)	1454 (5)
1975–79	135 (<1)	23 (<1)	158 (<1)
Age in 1998 (years), mean (range)^a^	42.9 (19–80)	39.1 (19–75)	42.6 (19–80)
Marital status, *n* (%)			
Single	2870 (11)	509 (20)	3379 (12)
Cohabitant/married	19 964 (79)	1683 (66)	21 647 (78)
Separated/divorced	2010 (8)	320 (12)	2330 (8)
Widow/widower	182 (1)	26 (1)	208 (1)
Missing	321 (1)	32 (1)	353 (1)
Number of children, *n* (%)			
0	4527 (18)	803 (31)	5330 (19)
1	3898 (15)	564 (22)	4462 (16)
2	8897 (35)	745 (29)	9642 (35))
3	5868 (23)	348 (14)	6216 (22)
≥4	1968 (8)	88 (3)	2056 (7)
Missing	189 (1)	22 (1)	211 (1)
Age at first child, mean (range)^a^	26.6 (16–75)	25.3 (16–42)	26.5 (16–75)
Educational level, *n* (%)			
Compulsory	3010 (12)	382 (15)	3392 (12)
Vocational training	10 412 (41)	562 (22)	10 974 (39)
Upper secondary	6003 (24)	853 (33)	6856 (25)
University/college	5736 (22)	745 (29)	6481 (23)
Missing	186 (1)	28 (1)	214 (1)
***Anthropometric***			
Height (cm), mean (range)^a^	180 (125–205)	167 (149–188)	179 (125–205)
Weight (kg), mean (range)^a^	83 (40–204)	66 (40–170)	82 (40–204)
BMI (kg/m^2^), *n* (%)			
12–18.4	49 (<1)	68 (3)	117 (<1)
18.5–24.9	11 072 (44)	1797 (70)	12 869 (46)
25.0–29.9	11 931 (47)	524 (20)	12 455 (45)
≥30.0	1947 (8)	136 (5)	2083 (7)
Missing	348 (1)	45 (2)	393 (1)
***Work history***			
No. of offshore jobs, mean (range)^a^	2.19 (1–8)	1.53 (1–8)	2.13 (1–8)
Employment duration (years), mean (range)^a^	11.0 (<1–40)	7.28 (<1–39.5)	10.6 (<1–40)
Main activity last position, *n* (%)			
Production	1812 (7)	151 (6)	1963 (7)
Drilling	3634 (14)	146 (6)	3780 (14)
Maintenance	12 657 (50)	273 (10)	12 930 (46)
Catering/office/administration	2955 (12)	1731 (68)	4686 (17)
Miscellaneous	4036 (16)	232 (9)	4268 (15)
Missing	253 (1)	37 (1)	290 (1)
Work schedule latest position, *n* (%)			
Daytime	12 698 (50)	1459 (57)	14 157 (51)
Night-time	1001 (4)	70 (3)	1071 (4)
Shift work	10 398 (41)	902 (35)	11 300 (40)
Missing	1250 (5)	139 (5)	1389 (5)
***Lifestyle***			
Smoking status, *n* (%)			
Never	7290 (29)	768 (30)	8058 (29)
Former	7527 (30)	607 (24)	8134 (29)
Current	9888 (39)	1133 (44)	11 021 (39)
Missing	642 (2)	62 (2)	704 (3)
Pack years, mean (range)^a^	11.6 (0–96.5)	9.3 (0–63.8)	11.4 (0–96.5)
Alcohol intake, *n* (%)^b^			
Never/rarely	1264 (5)	285 (11)	1549 (6)
Q1: 0.5–1.0 units/week	5481 (22)	1034 (40)	6515 (23)
Q2: 1.1–2.5 units/week	7230 (28)	636 (25)	7866 (28)
Q3: 2.6–4.0 units/week	4402 (17)	278 (11)	4680 (17)
Q4: 4.1–66.0 units/week	5795 (23)	216 (8)	6011 (22)
Missing	1175 (5)	121 (5)	1296 (5)
Red meat intake, *n* (%)^c^			
Q1: 0.0–7.0 times/week	5928 (23)	1112 (43)	7040 (25)
Q2: 7.1–9.5 times/week	5692 (22)	554 (22)	6246 (22)
Q3: 9.6–12.8 times/week	6439 (26)	502 (19)	6341 (25)
Q4: 12.9–59.8 times/week	6058 (24)	281 (11)	6339 (23)
Missing	1230 (5)	121 (5)	1351 (5)
Physical activity, n (%)^d^			
Never	6070 (24)	472 (19)	6542 (23)
1–3 times/month	7798 (31)	724 (28)	8522 (31)
1–2 times/week	6589 (26)	777 (30)	7366 (26)
3–4 times/week	3362 (13)	423 (17)	3785 (14)
5–7 times/week	1095 (4)	137 (5)	1232 (4)
Missing	433 (2)	37 (1)	470 (2)
Sunburns, *n* (%)^e^			
Never	5517 (22)	840 (33)	6357 (23)
1 time/year	16 224 (64)	1430 (56)	17 654 (63)
2–3 times/year	2565 (10)	203 (8)	2768 (10)
≥4 times/year	308 (1)	31 (1)	339 (1)
Missing	733 (3)	66 (2)	799 (3)
Sunbathing, *n* (%)^e^			
Never	1959 (8)	60 (2)	2019 (7)
1 week/year	7208 (28)	434 (17)	7642 (27)
2–3 weeks/year	9803 (39)	1187 (46)	10 990 (40)
≥4 weeks/year	5496 (22)	806 (32)	6302 (23)
Missing	881 (3)	83 (3)	964 (3)
Solarium use, *n* (%)^e^			
Never	11 759 (46)	331 (13)	12 090 (43)
Rarely	10 400 (41)	1523 (59)	11 923 (43)
1–2 times/month	1729 (7)	399 (16)	2128 (8)
≥3 times/month	888 (4)	264 (10)	1152 (4)
Missing	571 (2)	53 (2)	624 (2)
Sunscreen use, *n* (%)			
Never/rarely	10 137 (40)	495 (19)	10 632 (38)
Often	8436 (33)	737 (29)	9173 (33)
Almost always	6456 (26)	1308 (51)	7764 (28)
Missing	318 (1)	30 (1)	348 (1)

BMI, body mass index.

a Missing numbers in continuous variables: age at first child (*n* = 6190); height (*n* = 331); weight (*n *= 374); no. of offshore jobs (*n *= 278); employment duration (*n* = 1480); pack-years (*n *= 704) among former or current smokers.

b Summed units of beer (0.5 litre), wine (glass) or spirits (drink).

c Mean units of cold red meat (e.g. roast beef, boiled ham), paté, bacon, steaks, hamburgers and hot dogs consumed onshore and offshore during a week.

d Exercise the past year; minimum of 20 min with intensity that made you sweaty and short of breath.

e After age 20.

Each worker reported their job title, start date and stop date for up to eight jobs. The decision to limit the questionnaire to eight jobs per worker was based on an assumption in the project reference group (i.e. experts from the petroleum industry, unions and the Norwegian Petroleum Safety Authorities) that few workers would have more jobs. Only the first and last job were electronically readable from the questionnaires, meaning that title, start date and stop date for job 2 to job 7 were coded as free-text, and had to be extracted manually from the questionnaires. Less than 2 % reported eight jobs, which means the fraction of workers with more than eight jobs was small and that the loss of employment data due to this restriction was small. The self-reported job titles were mapped into 27 aggregate job categories based on correspondence with the project reference group.^18^^,^^19^ The work history data reported by the workers (start date, stop date, job) required systematic harmonization of overlapping employment records to avoid overestimating exposure before exposure linkage to job-exposure matrices (JEMs).^20^

### Development of job-exposure matrices (JEMs)

Table 4 gives an overview of the 18 JEMs that have been prepared specifically for the NOPW cohort, by type of exposure assessment, publication and exposed job categories. In 2005, a group at the University of Bergen started to develop expert-based JEMs where the aim was to identify and describe the degree of exposure to agents, mixtures or exposure situations with known and suspected carcinogenic potential among offshore workers on the Norwegian continental shelf who were employed 1970–2005.^18^^,^^19^ There has been a paucity of measurement data of known and suspected carcinogenic agents in the Norwegian offshore work environment, and most of the data available at the time of the JEM development were recorded after 1990.^21^ Hence, an expert-based approach was chosen for the development of the JEMs, where three university and five industry experts in occupational hygiene individually assessed the likelihood of exposure to 1836 combinations of carcinogens (*n *= 18), job categories (*n* = 27) and time periods (*n* = 4), resulting in the JEMs shown in Table 4. The JEMs and their development have been described in detail elsewhere.^18^^,^^19^^,^^22^^,^^23^

**Table 4.** Overview of job-exposure matrices available for the Norwegian Offshore Petroleum Workers (NOPW) cohort

Job-exposure matrices	Benzene	Mineral oil inhal.	Mineral oil skin	Crude oil skin	Oil mistvapour	Chlor. degreaser	Dichloro- methane	Asbestos	Cryst. silica	RCFs	Welding fumes	Diesel exhaust	Nickel	Chrome IV	Inorganic lead	Formal- dehyde	Exposure as painter	Ionizing radiation
**Type of exposure assessment (X)**																		
Expert-based	X	X	X	X		X	X	X	X	X	X	X	X	X	X	X	X	X
Semi-quantitative	X							X										
Quantitative					X													
Measurement data used	X				X			X										
**JEM publication (X)**																		
Steinsvåg *et al*., 2005	X	X	X	X		X	X	X	X	X	X	X	X	X	X	X	X	X
Steinsvåg *et al*., 2007	X	X	X	X		X	X	X	X	X	X	X	X	X	X	X	X	X
Bråtveit *et al*., 2011	X				X			X										
**Exposed job-categories (X)**																		
Production																		
Process technicians	X	X	X	X		X		X					X	X	X	X		X
Control room operators																		
Laboratory engineers	X			X		X	X									X		
Drilling																		
Drill floor crew		X	X	X	X	X		X	X			X			X			
Shale shaker operators		X	X	X	X	X		X	X			X				X		
Derrick employees		X	X	X	X	X		X	X			X						
Drillers			X	X	X	X		X							X			X
Well service crew			X	X	X	X		X	X			X						
MWD and mud loggers/engineers		X	X	X	X	X												X
Maintenance																		
Electricians		X		X		X		X	X	X								
Electric instrument technicians	X	X		X		X		X										
Radio employees																		
Non-destructive testing	X																	X
Plumbers and piping engineers	X	X		X		X		X	X	X	X		X	X				
Welders	X			X		X		X			X		X	X	X			
Sheet metal workers	X			X		X		X			X		X	X	X			
Mechanics	X	X	X	X		X	X	X			X		X	X	X			
Machinists	X	X	X			X		X				X					X	
Turbine operators, hydraulics technicians		X	X			X		X										
Deck crew	X	X	X	X		X		X			X	X	X	X	X	X	X	X
Industrial cleaner	X	X	X	X		X		X	X									
Surface treatment (painters)				X		X	X	X	X	X			X	X	X		X	
Scaffold crew	X			X				X	X								X	
Insulators	X			X				X	X	X								
Catering/office/administration																		
Catering workers						X												
Chefs																		
Health, office administration																		

Chlor., chlorinated; inhal., inhale; cryst., crystalline; MWD, measure while drilling; RCF, refractory ceramic fibres.

In 2010–11, the JEMs for benzene, asbestos and oil mist/vapour were refined with more measurement data and a new methodological approach, in order to obtain exposure estimates that: (i) more clearly captured the contrasts in exposure between job categories and time periods; and (ii) were based on exposure determinants related to performed work-tasks, rather than probability for exposure at the job-category level.^21^ As indicated by type of exposure assessment in Table 4, two different refinement strategies were chosen for the three agents. For benzene and asbestos, the paucity of measurement data prompted a semi-quantitative and task-oriented strategy.^24^ For oil mist/vapour, however, measurement data during offshore drilling in Norway 1979–2004 were published by Steinsvåg *et al*.,^25^ who concluded that exposure to oil mist/vapour declined over time, and that exposure levels were associated with rig type, mud temperature, technical control measures, type and viscosity of the base oil, work area and season. Subsequently, these measurement data were used for development of the oil mist/vapour JEM with quantitative exposure estimates for drilling workers (Table 4).^21^ Details of the JEM refinement have been published elsewhere.^15^^,^^16^^,^^21^

## What has been found? Key findings and publications

The studies that have been published from the cohort thus far have been of three types of epidemiological study design: cross-sectional studies (*n *= 2),^26^^,^^27^ cohort studies (*n* = 2)^12^^,^^14^ and case-cohort studies (*n* = 3).^15–17^ In addition, there was one methodological paper on harmonization of overlapping employment records.^20^ The cross-sectional studies used only data from the baseline questionnaire and described education, onshore occupations and factors associated with self-reported exposures in the offshore work environment. The cohort studies were linked to the CRN for prospective analyses of cancer risk, and SIRs were calculated for comparison with the Norwegian background population. The case-cohort design was used because work history data from job 2 to job 7 had to be extracted manually in order to obtain full work histories for each worker. To limit costs, this was extracted only for a random sample of the cohort (i.e. subcohort) and for all cancer cases based on a stratified case-cohort design.^28^ Simulation studies have confirmed that relative risk estimates from stratified case-cohort studies are similar to those produced by traditional cohort studies.^29^

In the first linkage (follow-up 1999–05), 695 male cancer cases were identified and the overall cancer incidence was close to that expected. Indications of excess risks were found for acute myeloid leukaemia (AML), (SIR 2.00, 95% CI: 0.97–3.72) and mesothelioma (SIR 2.18, 95% CI: 0.89–4.55).^2^^,^^14^ Among women, 78 cases were identified and excesses were found for the overall cancer incidence (SIR 1.31, 95% CI: 1.04–1.64) and for melanoma (SIR 2.75, 95% CI: 1.42–4.81).^2^ The first cross-sectional study showed that 59% had work experience before starting their offshore career and that 32% reported being employed outside the offshore sector at baseline. Vocational training was the most frequently reported educational level (39%).^26^

In 2014, the NOPW cohort was merged with the register-based cohort from the NREE (described above), resulting in a total of 41 000 workers including 2191 incident cancer cases identified between 1999 and 2009.^12^ In males, increased risks were seen for cancer of the urinary bladder (SIR 1.25, 95% CI: 1.05–1.49), the lung (SIR 1.14, 95% CI: 1.00–1.30) and the pleura (SIR 2.56, 95% CI; 1.58–3.91. A possible excess of kidney cancer was also observed (SIR 1.13, 95% CI: 0.90–1.39). In females, excesses were observed for overall cancer incidence (SIR 1.17, 95% CI: 1.02–1.34), lung cancer (SIR 1.69, 95% CI: 1.03–2.61), AML (SIR 5.29, 95% CI: 1.72–12) and melanoma (SIR 2.13, 95% CI: 1.41–3.08).

In 2015, a cross-sectional study was conducted to identify predictors of self-reported exposures. We found that holding a non-supervisory position, working shifts, being employed in the early period of the offshore industry and having only compulsory education increased the probability of reporting frequent exposure (e.g. vapours from mud, drilling/processing chemicals and solvents, exhaust fumes, natural gas).^15^

During 2015–17, three case-cohort studies were conducted to estimate cause-specific cancer risk. For benzene exposure, we found evidence for dose-response relationships with AML and multiple myeloma (*P*_trends_ 0.052 and 0.024, respectively), and suggestively with chronic lymphocytic leukemia (*P*_trend_ 0.094).^15^ For melanomas and non-melanomas of the forearm and hand, cumulative and duration metrics of benzene or of crude oil exposure showed *P*_trends_ <0.05.^16^ For other anatomical sites, we observed an increased skin cancer risk associated with sunburn frequency and risk of melanoma and non-melanoma skin cancer (*P*_trends_ <0.05 and <0.01, respectively).^17^

Table 2 shows SIRs of the latest linkage. Overall increased cancer risks were seen for both males (SIR 1.07, 95% CI: 1.04–1.11) and females (SIR 1.13, 95% CI: 1.01–1.26). Among males, elevated risk was found for pleura (SIR 2.38, 95% CI: 1.63–3.36), breast (SIR 2.18, 95% CI: 1.13–3.81) and prostate (SIR 1.20, 95% CI: 1.14–1.27). In females, risks of melanoma (SIR 1.62, 95% CI: 1.11–2.29) and AML (SIR 3.76, 95% CI: 1.22–8.78) were increased.

## What are the main strengths and weaknesses?

A major asset of the NOPW cohort is the comprehensive questionnaire with extensive information on work history and potential confounding factors. Work history was recorded also for onshore periods before and after the offshore career, and in off-duty periods. Although these data are self-reported with the inherent potential of information bias, self-reporting of work histories has been found to be accurate and robust.^30–33^ Since all JEMs are developed by an independent group of industrial hygienists, and the work history was reported before diagnosis, we consider it as likely that neither exposure to specific agents nor case status affected the workers’ reporting. Thus, misclassification was most likely non-differential, which would result in attenuation of the effect estimate of the higher exposure category in crude age-adjusted models, whereas in multivariable models, non-differential misclassification may bias the effect estimates both away and towards the null.^34^ Due to the 11-digit PIN, linkage can be done with any population-based register in Norway, including the CRN, the Cause of Death Registry and the National Population Register (data on year of death/emigration) which enables precise calculation of person-time.

An important limitation is that data collection has only been performed once, in 1998. Hence, work histories only cover the period before and up to baseline: 1965–98. Thus, it is not possible to examine the effect of occupational exposure during the period of cancer follow-up from 1999 onwards. Moreover, no biological samples or clinical measurements have been collected, which hampers examination of molecular hypotheses.

## Can I get hold of the data? Where can I find out more?

The data are held by the CRN. Requests for data sharing/case pooling may be directed to principal investigator Dr Tom K Grimsrud [tom.k.grimsrud@kreftregisteret.no]. Participation in the NOPW cohort studies is based on informed consent, which must be considered whenever use of data deviates from the original plans. Moreover, the research file uses data derived from state government registries, which deliver data under licence from regional committees for research ethics and data custodians. Thus, any requests to share these data will be subject to formal considerations and approval must be obtained from each data source. Background information on the study, the scientific team and study progress is available through the study website [https://www.kreftregisteret.no/en/Research/Projects/cancer-among-offshore-workers-in-norway/].

**Profile in a nutshell**

- The Norwegian Offshore Petroleum Workers (NOPW) cohort was set up in 1998 for prospective follow-up of nearly 28 000 offshore workers for cancer incidence and cause-specific mortality; over 4000 cancer cases were identified by 2017.
- The NOPW cohort is to our knowledge unique in consisting solely of offshore petroleum workers, which offers possibilities of gaining new insight into this part of upstream petroleum industry.
- A range of job-exposure matrices has been developed specifically for the NOPW cohort, enabling studies of occupational exposures and cancer risk and mortality.
- Due to the unique personal identification numbers, linkage can be done with national health registries, with complete follow-up and high-quality endpoints.

## Supplementary Data

Supplementary data are available at *IJE* online.

## Funding

This work was funded by grant 280537 from the Research Council of Norway’s PETROMAKS2 programme to the Cancer Registry of Norway (J.S.S., R.B., F.C.L., N.S. and T.K.G.). J.K. was funded by grant 280904 from the Research Council of Norway’s PETROMAKS2 programme. N.R., Q.L., D.T.S. and M.C.F. were funded by the Intramural Research Program of the Division of Cancer Epidemiology and Genetics, at the U.S. National Cancer Institute of the National Institutes of Health.

## Supplementary Material

dyaa107_Supplementary_Data
