# Supplementary material for: Cohort Profile: Norwegian Offshore Petroleum Workers (NOPW) Cohort
Source: Int J Epidemiol. 2020 Sep 2;50(2):398–9. doi: 10.1093/ije/dyaa107 (PMC8128454; doi:10.1093/ije/dyaa107)
Supplement: dyaa107_Supplementary_Data [file dyaa107_supplementary_data.zip › ije-2019-09-1231-File007.docx]

**SUPPLEMENTAL MATERIAL**

**Title:** Cohort Profile: The Norwegian Offshore Petroleum Workers (NOPW) Cohort

**Authors:** Jo S Stenehjem^*1,2,3^, Ronnie Babigumira^2^, H Dean Hosgood^4^, Marit B Veierød^1^, Sven Ove Samuelsen^5^, Magne Bråtveit^6^, Jorunn Kirkeleit^6,7^, Nathaniel Rothman^8^, Qing Lan^8^, Debra T Silverman^8^, Melissa C Friesen^8^, Trude E Robsahm^2^, Kristina Kjærheim^2^, Bettina K Andreassen^2^, Nita K Shala^2^, Fei-Chih Liu^2^, Leif-Åge Strand^9^, Tom K Grimsrud^†2^

^1^Oslo Centre for Biostatistics and Epidemiology, Department of Biostatistics, University of Oslo, Oslo, Norway

^2^Department of Research, Cancer Registry of Norway, Oslo, Norway

^3^Division of Emergencies and Critical Care, Oslo University Hospital, Norway

^4^Department of Epidemiology and Population Health, Albert Einstein College of Medicine, The Bronx, NY, United States

^5^Department of Mathematics, University of Oslo, Oslo, Norway

^6^Department of Global Public Health and Primary Care, University of Bergen, Bergen, Norway

^7^Department of Occupational Medicine, Haukeland University Hospital, Bergen, Norway

^8^Occupational and Environmental Epidemiology Branch, Division of Cancer

Epidemiology and Genetics, National Cancer Institute, Bethesda, MD, United States

^9^Norwegian Armed Forces Joint Medical Services, Sessvollmoen, Norway.

^*^Corresponding author

^†^Principal Investigator

**Content**

- Table S1: Cohort establishment

| **Table S1.** Cohort establishment | | |
| --- | --- | --- |
| **Subject Groups** |  | **No. of Subjects** |
| Roster of possible offshore workers^a^ |  | 61 339 |
| Dead before September 1998 | – | 1572 |
| Emigrated before September 1998 | – | 2438 |
| Eligible for questionnaire | = | 57 329 |
| Non-respondents | – | 21 871 |
| Respondents, never worked offshore | – | 7249 |
| Missing address | – | 222 |
| Respondents, worked offshore | = | 27 987 |
| Not meeting criteria^b^ | – | 68 |
| Excluded, missing personal ID number | – | 2 |
| Offshore workers included in the cohort | = | 27 917 |
| ^a^Compiled from lists from oil companies, educational institutions, trade unions, and other sources. | | |
| ^b^Worked on ships with no drilling or production activity: e.g. supply, lifting, seismic, and pipe laying vessels. | | |
