# Supplementary material for: Cohort Profile: Norwegian Offshore Petroleum Workers (NOPW) Cohort
Source: Int J Epidemiol. 2020 Sep 2;50(2):398–9. doi: 10.1093/ije/dyaa107 (PMC8128454; doi:10.1093/ije/dyaa107)
Supplement: dyaa107_Supplementary_Data [file dyaa107_supplementary_data.zip › ije-2019-09-1231-File008.pdf]

Survey on working environment, lifestyle, and cancer risk among Norwegian offshore workers

Questionnaire translated from Norwegian

BACKGROUND QUESTIONS

Have you been working 20 days or more on a stationary or moveable platform (installation) on the Norwegian continental shelf?

☐ Yes. Please answer all questions and return the questionnaire
☐ No. Please answer question 1 *only* and return the questionnaire

---

1. What year were you born? 19

2. Marital status. Tick *one* category only.

☐ Single
☐ Cohabitant/married
☐ Separated/divorced
☐ Widow/widower

3. a) Do you have children?

☐ Yes
☐ No → *Continue with question 4*

3. b) How many children do you have? ☐ number of children

3. c) How old were you when your first child was born? ☐ years of age

4. What is your highest level of completed education? Tick *one* category only.

1 ☐ Primary and lower secondary (compulsory)
2 ☐ Folk high school (one-year boarding school)
3 ☐ Upper secondary
4 ☐ Vocational training
5 ☐ University/college

5. What is your current employment status? Tick *one* category only.

☐ Employed in work offshore or onshore/offshore tours
☐ Employed in other work
☐ Student
☐ Unemployed/on leave
☐ On sick leave/rehabilitation
☐ Recipient of disability benefit
☐ Retired

CURRENT OR LAST POSITION OFFSHORE (questions 6–15)

6. What is your current or was your last position offshore?
If you have or had a combined position, please report as "CRANE OPERATOR/MECHANIC", "ROUSTABOUT/HELICOPTER WATCH", and so on.

Position (type of occupation) [redacted]

7. What year did you start in this position? (Questions on any earlier positions offshore follow on the subsequent pages)
19

8. For how long did you stay in this position? years and months

9. In which of the following categories does this position belong? Tick *one* category only. For combined positions, Tick the category corresponding to the main area of work.

☐ Drilling/well maintenance
☐ Office/administration/health
☐ Catering/cleaning
☐ Maintenance/inspection/deck/construction
☐ Production/process
☐ Maritime positions

10. Do you or did you have any managerial or supervisory responsibility in this position?

☐ Yes
☐ No

11. What kind of work schedule do you have or did you have in this position? Tick for the schedule you maintained for the longest time period:

☐ Daytime
☐ Nighttime
☐ Shiftwork

12. What type of company do you or did you work for in this position?

☐ Operator company
☐ Contractor company

13. What is or was the name of the installation you work or worked on in this position? If you have been working on several installations, name the one you worked on for the longest time period:

14. What kind of installation is or was this?

☐ Stationary
☐ Movable

15. a) In this position, do you work or did you work...

☐ Both offshore and land-based (combined onshore/offshore)
☐ Full-time offshore. *Jump to question 16*

15. **b) Report the average proportion of offshore work in this position?**

☐ ca ¾ of the time
☐ ca ½ of the time
☐ ca ¼ of the time

16. **Have you held more than one position offshore?** A "position" is defined as the time you had the same occupational title with the same employer. If you, for example, changed from a drill floor crew to a derrickman, you start in a new position. If you were working full-time offshore in one period, and held a combined position (onshore and offshore) in another period, these two positions are defined as two separate positions.

☐ Yes → *Continue with question 17*
☐ No → *Continue with question 29*

EARLIER POSITIONS OFFSHORE (questions 17-28)

17. **What year did you first start in a position offshore?** 19

18. **What kind of offshore position did you hold at that time?** If you had a combined position, please report as "CRANE OPERATOR/MECHANIC", "ROUSTABOUT/HELICOPTER WATCH", and so on.

Position (type of occupation)

19. **For how long did you stay in this position?** years and months

20. **In which of these categories does the position belong?** Tick *one* category only. For combined positions, Tick the category corresponding to the main area of work.

☐ Drilling/well maintenance
☐ Office/administration/health
☐ Catering/cleaning
☐ Maintenance/inspection/deck/construction
☐ Production/process
☐ Maritime positions

21. **Did you have any managerial or supervisory responsibility in this position?**

☐ Yes
☐ No

22. **What kind of work schedule did you have in this position?** Tick for the work schedule you maintained for the longest time period:

☐ Daytime
☐ Nighttime
☐ Shiftwork

23. **What type of company did you work for in this position?**

☐ Operator company
☐ Contractor company

24. What is or was the name of the platform/installation you worked on in this position? If you were working on several installations, name the one you worked on for the longest time period:

25. What kind of installation was this?

☐ Stationary
☐ Movable

26. a) In this position, do you work or did you work...

☐ Both offshore and land-based (combined onshore/offshore)
☐ Full-time offshore. *Continue with question 27*

26. b) Report the proportion of offshore work in this position?

☐ ca ¾ of the time
☐ ca ½ of the time
☐ ca ¼ of the time

27. Have you held more than one position offshore?

☐ Yes. → *Continue with question 28*
☐ No. → *Continue with question 29*

28. In this section, we ask you to state any other position you have held offshore. Also include work in the British offshore sector and other offshore sectors. Fill in the tables below in the same way as you responded to question 6–15 and 17–26. Please use one table for each position held, although you may merge several short-term positions, of the same type, into one table.

Example: You worked for the drilling company Maersk on the installation Maersk Guardian. You worked as a drill floor worker from May 1992 until you started as a derrick man in October 1995. You were not a manager, and worked mainly daytime. Please report as follows (red text):						
Position: DRILL FLOOR WORKER						
Category: ☒ Drilling/well maintenance ☐ Production/process		☐ Maintenance/inspection/deck/construction ☐ Catering/cleaning			☐ Office/administration/health ☐ Maritime positions	
Start year	For how long did you stay in this position?	Manager/supervisor	Work schedule	Type of company	Sector	Installation
19 92	3 years 5 months	☐ Yes ☒ No	☒ Day ☐ Night ☐ Shift	☐ Operator ☒ Contractor	☒ Norwegian ☐ British ☐ Other	☐ Stationary ☒ Movable
Installation name: MAERSK GUARDIAN						
Proportion of work hours offshore in this position: ☒ Full-time ☐ ca ¾ of the time ☐ ca ½ of the time ☐ ca ¼ of the time or less						

Fill in table 1–6 as shown in the example:

Table 1

Position:						
Category: ☐ Drilling/well maintenance ☐ Production/process		☐ Maintenance/inspection/deck/construction ☐ Catering/cleaning			☐ Office/administration/health ☐ Maritime positions	
Start year	For how long did you stay in this position?	Manager/supervisor	Work schedule	Type of company	Sector	Installation
19	years months	☐ Yes ☐ No	☐ Day ☐ Night ☐ Shift	☐ Operator ☐ Contractor	☐ Norwegian ☐ British ☐ Other	☐ Stationary ☐ Movable
Installation name:						
Proportion of work time offshore in this position: ☐ Full-time ☐ ca ¾ of the time ☐ ca ½ of the time ☐ ca ¼ of the time						

Table 2

Position: ....

Category: ☐ Drilling/well maintenance ☐ Production/process ☐ Maintenance/inspection/deck/construction ☐ Catering/cleaning ☐ Office/administration/health ☐ Maritime positions

Start year	For how long did you stay in this position?	Manager/supervisor	Work schedule	Type of company	Sector	Installation
19	years months	☐ Yes<br>☐ No	☐ Day<br>☐ Night<br>☐ Shift	☐ Operator<br>☐ Contractor	☐ Norwegian<br>☐ British<br>☐ Other	☐ Stationary<br>☐ Movable

Installation name: ....

Proportion of work time offshore in this position: ☐ Full-time ☐ ca ¾ of the time ☐ ca ½ of the time ☐ ca ¼ of the time

Table 3

Position: ....

Category: ☐ Drilling/well maintenance ☐ Production/process ☐ Maintenance/inspection/deck/construction ☐ Catering/cleaning ☐ Office/administration/health ☐ Maritime positions

Start year	For how long did you stay in this position?	Manager/supervisor	Work schedule	Type of company	Sector	Installation
19	years months	☐ Yes<br>☐ No	☐ Day<br>☐ Night<br>☐ Shift	☐ Operator<br>☐ Contractor	☐ Norwegian<br>☐ British<br>☐ Other	☐ Stationary<br>☐ Movable

Installation name: ....

Proportion of work time offshore in this position: ☐ Full-time ☐ ca ¾ of the time ☐ ca ½ of the time ☐ ca ¼ of the time

Table 4

Position: ....

Category: ☐ Drilling/well maintenance ☐ Production/process ☐ Maintenance/inspection/deck/construction ☐ Catering/cleaning ☐ Office/administration/health ☐ Maritime positions

Start year	For how long did you stay in this position?	Manager/supervisor	Work schedule	Type of company	Sector	Installation
19	years months	☐ Yes<br>☐ No	☐ Day<br>☐ Night<br>☐ Shift	☐ Operator<br>☐ Contractor	☐ Norwegian<br>☐ British<br>☐ Other	☐ Stationary<br>☐ Movable

Installation name: ....

Proportion of work time offshore in this position: ☐ Full-time ☐ ca ¾ of the time ☐ ca ½ of the time ☐ ca ¼ of the time

Table 5

Position: ....

Category: ☐ Drilling/well maintenance ☐ Production/process ☐ Maintenance/inspection/deck/construction ☐ Catering/cleaning ☐ Office/administration/health ☐ Maritime positions

Start year	For how long did you stay in this position?	Manager/supervisor	Work schedule	Type of company	Sector	Installation
19	years months	☐ Yes<br>☐ No	☐ Day<br>☐ Night<br>☐ Shift	☐ Operator<br>☐ Contractor	☐ Norwegian<br>☐ British<br>☐ Other	☐ Stationary<br>☐ Movable

Installation name: ....

Proportion of work time offshore in this position: ☐ Full-time ☐ ca ¾ of the time ☐ ca ½ of the time ☐ ca ¼ of the time

Table 6

Position: ....

Category: ☐ Drilling/well maintenance ☐ Production/process ☐ Maintenance/inspection/deck/construction ☐ Catering/cleaning ☐ Office/administration/health ☐ Maritime positions

Start year	For how long did you stay in this position?	Manager/supervisor	Work schedule	Type of company	Sector	Installation
19	years months	☐ Yes<br>☐ No	☐ Day<br>☐ Night<br>☐ Shift	☐ Operator<br>☐ Contractor	☐ Norwegian<br>☐ British<br>☐ Other	☐ Stationary<br>☐ Movable

Installation name: ....

Proportion of work time offshore in this position: ☐ Full-time ☐ ca ¾ of the time ☐ ca ½ of the time ☐ ca ¼ of the time

WORKING ENVIRONMENT OFFSHORE

29. During your work offshore, do you or did you experience any of the exposures mentioned below? If yes, report the proportion of the working hours you experienced such exposure. For each question, please Tick the one of the six categories that fits best: almost all of the working hours, ca ¾ of the working hours, ca ½ of the working hours, ca ¼ of the working hours, rarely, and never.

Report the proportion of the working hours, during your current or last position, you were exposed to:

	Tick *one* box for each row					
	Almost all the time	¾ of the time	½ of the time	¼ of the time	Rarely	Never
Heavy physical work	☐	☐	☐	☐	☐	☐
Sedentary work	☐	☐	☐	☐	☐	☐
Noise	☐	☐	☐	☐	☐	☐
Passive smoking in working- and/or break room	☐	☐	☐	☐	☐	☐
Skin contact with detergents and/or disinfectants	☐	☐	☐	☐	☐	☐
Skin contact with oil or diesel	☐	☐	☐	☐	☐	☐
Oil vapour from shaker and other mud cleaning	☐	☐	☐	☐	☐	☐
Exhaust fumes	☐	☐	☐	☐	☐	☐
Natural gas (from sampler, water cleaning plant and vent. systems etc.)	☐	☐	☐	☐	☐	☐
Dust from mixing of drilling chemicals	☐	☐	☐	☐	☐	☐
Vapour from mixing of drilling chemicals	☐	☐	☐	☐	☐	☐
Chemicals for water injection and process	☐	☐	☐	☐	☐	☐
Dust from sandblasting and grinding	☐	☐	☐	☐	☐	☐
Welding fumes	☐	☐	☐	☐	☐	☐
Solvent vapour (painting, cleaning, degreasing etc.)	☐	☐	☐	☐	☐	☐
Cooking fumes	☐	☐	☐	☐	☐	☐
Radioactive material (radiography, logging, low-radioactive coating etc.)	☐	☐	☐	☐	☐	☐

30. Du you hold, or have you ever held a position offshore with full shift rotation with off duty periods with duration of 2–4 weeks?

☐ Yes. → *Continue with question 31*
☐ No. → *Continue with question 34*

OFF-DUTY PERIODS

In this section we ask you to report various activities in your off duty periods. If you do not work offshore with full rotation anymore, please report activities from when you were engaged in rotation with off duty periods with duration of 2–4 weeks.

31. What was the duration of your last off-duty period?

☐ 2 weeks
☐ 3 weeks
☐ 4 weeks

32. How many times during the last off-duty period did you:

	Tick *one* box for each row				
	None	1–2 times	3–5 times	6–9 times	10 times or more
Practice long hiking or cross country skiing	☐	☐	☐	☐	☐
Attend a cultural or sporting event..	☐	☐	☐	☐	☐
Visit a café or restaurant...	☐	☐	☐	☐	☐
Visit a pub/bar or discotheque...	☐	☐	☐	☐	☐
Participate in a sports or exercise event................................	☐	☐	☐	☐	☐
Participate in a musical, organizational, and charity event...	☐	☐	☐	☐	☐

33. During the off-duty periods of the last year you were working offshore, please report the proportion of time you usually spent on:

	Tick *one* box for each row			
	No time	Little time	Much time	Almost all time
Staying at home with my children...	☐	☐	☐	☐
Resting and recovering..	☐	☐	☐	☐
Holiday travels..	☐	☐	☐	☐
Courses and training..	☐	☐	☐	☐
Maintenance work on house, cabin etc......................................	☐	☐	☐	☐
Maintenance work on car/motorcycle/boat..............................	☐	☐	☐	☐
Paid work (please specify in question 35)................................	☐	☐	☐	☐

34. During your professional career, have you ever held a position outside the offshore sector for more than four months?

Disregard positions where you combined onshore and offshore work, but please report onshore positions during off-duty periods.

☐ Yes. → *Continue with question 35*
☐ No. → *Continue with question 36a*

POSITIONS BEFORE/AFTER OFFSHORE AND IN OFF-DUTY PERIODS

35. What type of positions outside the offshore sector have you held? Please classify each position according to the corresponding industry sectors:

1 = Shipping, *bridge/deck*
2 = Shipping, *machinist*
3 = Shipping, *other*
4 = Fishing industry
5= Farming/forestry
6 = Chemical industry
7 = Heavy industry/works/mech. ind.
8 = Other industry
9 = Building and construction
10 = Painting/surface treatment
11 = Eletrical occupations
12 = Health/social services
13 = Trade/office services
14 = Hotel and restaurant
15 = Housework
16 = Academics
17 = Military
18 = Transport
19 = Other (specify in table)

In the table below, please type the *number* corresponding to the industrial sector for each position, and add information on start and stop for this position, and specify occupational title or type of work. You may merge several short-term positions within the same sector when one position followed the other, and no more than a one-year gap was in-between. State whether you worked full-time or part-time, or if you worked during off-duty periods.

Example: You have been working full-time as a nurse on a hospital or in a nursing home from 1978 until you started offshore in May 1991. Subsequently, you worked part-time in nursing home in the off duty periods. This should be stated in the first two rows of the table as follows (red text):

Ind. sector no.	Start year	Stop year	Occupational title/type of work	You worked: Full-time	Part-time	In off duty periods
12	1978	1991	NURSE	☒	☐	☐
12	1991	19	NURSE	☐	☐	☒
	19	19		☐	☐	☐
	19	19		☐	☐	☐
	19	19		☐	☐	☐
	19	19		☐	☐	☐
	19	19		☐	☐	☐
	19	19		☐	☐	☐
	19	19		☐	☐	☐
	19	19		☐	☐	☐
	19	19		☐	☐	☐

LIFESTYLE AND DIET

36. a) Do you ever drink alcohol?

☐ Yes. → *Continue with question 36b*

☐ No, I have quit drinking alcohol → *Continue with question 37a*

☐ No, I am teetotal → *Continue with question 37a*

36. b) If yes, how was your average alcohol consumption last year?

Tick *one* box for each row

	Never/ rarely	1-3 pr month	1 pr week	2-4 pr week	5-8 pr week	9-14 pr week	15-19 pr week	20+ pr week
Beer (0.5 liters)	☐	☐	☐	☐	☐	☐	☐	☐
Wine (glass)	☐	☐	☐	☐	☐	☐	☐	☐
Spirits (drinks)	☐	☐	☐	☐	☐	☐	☐	☐

A large bottle of vodka, whisky etc = 20 drinks, a large bottle of 60% = 30 drinks

37. a) Have you ever smoked on a daily basis?

☐ Yes. → *Continue with question 37b*

☐ No, I have quit drinking alcohol → *Continue with question 38*

37. b) If you have smoked on a daily basis, please report how many cigarettes or pipes of tobacco you consumed daily for each age-period of your life.

No. of cigarettes or pipes pr. day

Age	0	1-4	5-9	10-14	15-19	20-24	25-29	30+
15-19 yrs	☐	☐	☐	☐	☐	☐	☐	☐
20-29 yrs	☐	☐	☐	☐	☐	☐	☐	☐
30-39 yrs	☐	☐	☐	☐	☐	☐	☐	☐
40-49 yrs	☐	☐	☐	☐	☐	☐	☐	☐
50-59 yrs	☐	☐	☐	☐	☐	☐	☐	☐
60+ yrs	☐	☐	☐	☐	☐	☐	☐	☐

37. c) What type of tobacco do you or did you smoke?

Tick of *one* box

☐ Cigarettes

☐ Roll-your-own tobacco

☐ Cigarettes and roll-your-own tobacco

☐ Pipe tobacco

38. How many times a year did you become sunburned to the extent that your skin became red and blistered and later peeled off?

Age	Never	Max. once a year	2–3 times a year	4–5 times a year	16 times or more a year
Before 20 yrs	☐	☐	☐	☐	☐
After 20 yrs	☐	☐	☐	☐	☐

39. How often have you been sunbathing in a solarium?

Age	Never	Rarely	1–2 times a month	3–4 times a month	More than once a week
Before 20 yrs	☐	☐	☐	☐	☐
After 20 yrs	☐	☐	☐	☐	☐

40. How many weeks per year do you sunbathe outdoors?

Age	Never	1 week	2–3weeks	4–5 weeks	7 weeks or more
Before 20 yrs	☐	☐	☐	☐	☐
After 20 yrs	☐	☐	☐	☐	☐

41. Do you use sunscreen lotion/cream?

☐ Rarely/never
☐ Often
☐ Almost always

---

42. Outside working hours, how often have you been exercising for a minimum of 20 minutes in a way that made you sweaty and short of breath?

	Never/ rarely	1–3 times a month	1–2 times a week	3–4 times a week	5–6 times a week	Every day
During the last year	☐	☐	☐	☐	☐	☐
10 years ago	☐	☐	☐	☐	☐	☐

---

43. How many cups or glasses do you usually drink of these beverages?

	Tick *one* box for each row						
	Never/ rarely	1–6 a week	1 a day	2–3 a day	4–5 a day	6–7 a day	8+ a day
Coffee	☐	☐	☐	☐	☐	☐	☐
Tea	☐	☐	☐	☐	☐	☐	☐
Juice	☐	☐	☐	☐	☐	☐	☐
Milk	☐	☐	☐	☐	☐	☐	☐
Light-soda, artificial sweetener only	☐	☐	☐	☐	☐	☐	☐
Soda, non-alcoholic beer, mineral water	☐	☐	☐	☐	☐	☐	☐

---

44. Diet at home. During the last week you were at home, how many times the did you eat any of these food items?

Tick *one* box for each row

	Did not eat	1–2 times a week	3–4 times a week	5–6 times a week	Every day	More than once a day
Carrot, raw or cooked	☐	☐	☐	☐	☐	☐
Other vegetables, single or mixed in salads etc.	☐	☐	☐	☐	☐	☐
Mayonnaise salads (coleslaw, shrimp, Waldorf etc)	☐	☐	☐	☐	☐	☐
Potato, boiled or baked	☐	☐	☐	☐	☐	☐
French fries, fried potato	☐	☐	☐	☐	☐	☐
Orange, clementine, mandarin	☐	☐	☐	☐	☐	☐
Other fruits and berries (except jam)	☐	☐	☐	☐	☐	☐
Cream, sour cream, whipped cream	☐	☐	☐	☐	☐	☐
Roast beef, boiled ham, cured ham, salami	☐	☐	☐	☐	☐	☐
Pâté	☐	☐	☐	☐	☐	☐
Egg (scrambled, omelet, boiled)	☐	☐	☐	☐	☐	☐
Bacon	☐	☐	☐	☐	☐	☐
Beef, t-bone steak	☐	☐	☐	☐	☐	☐
Other steaks	☐	☐	☐	☐	☐	☐
Bernaise sauce, dressing	☐	☐	☐	☐	☐	☐
Rissoles, hamburgers, Vienna steak	☐	☐	☐	☐	☐	☐
Hot dogs, sausages	☐	☐	☐	☐	☐	☐
Smoked salmon, mackerel	☐	☐	☐	☐	☐	☐
Fried fish	☐	☐	☐	☐	☐	☐
Other fish	☐	☐	☐	☐	☐	☐

45. Diet offshore. During the last week you were offshore, how many times did you eat any of these food items? If you do not work offshore presently, please report a typical week from your last position offshore.

Tick *one* box for each row

	Did not eat	1–2 times a week	3–4 times a week	5–6 times a week	Every day	More than once a day
Carrot, raw or cooked	☐	☐	☐	☐	☐	☐
Other vegetables, single or mixed in salads etc.	☐	☐	☐	☐	☐	☐
Mayonnaise salads (coleslaw, shrimp, Waldorf etc)	☐	☐	☐	☐	☐	☐
Potato, boiled or baked	☐	☐	☐	☐	☐	☐
French fries, fried potato	☐	☐	☐	☐	☐	☐
Orange, clementine, mandarin	☐	☐	☐	☐	☐	☐
Other fruits and berries (except jam)	☐	☐	☐	☐	☐	☐
Cream, sour cream, whipped cream	☐	☐	☐	☐	☐	☐
Roast beef, boiled ham, cured ham, salami	☐	☐	☐	☐	☐	☐
Pâté	☐	☐	☐	☐	☐	☐
Egg (scrambled, omelet, boiled)	☐	☐	☐	☐	☐	☐
Bacon	☐	☐	☐	☐	☐	☐
Beef, t-bone steak	☐	☐	☐	☐	☐	☐
Other steaks	☐	☐	☐	☐	☐	☐
Bernaise sauce, dressing	☐	☐	☐	☐	☐	☐
Rissoles, hamburgers, Vienna steak	☐	☐	☐	☐	☐	☐
Hot dogs, sausages	☐	☐	☐	☐	☐	☐
Smoked salmon, mackerel	☐	☐	☐	☐	☐	☐
Fried fish	☐	☐	☐	☐	☐	☐
Other fish	☐	☐	☐	☐	☐	☐

A

46. Do you usually take food supplements? You may tick several boxes.

- ☐ I do not take food supplements
- ☐ I take cod-liver oil
- ☐ I take Vitamin C
- ☐ I take multivitamin supplements
- ☐ I take fish-oil capsules
- ☐ Other:

47. Have you been taking sleeping pills?

- ☐ No, never
- ☐ Yes, occasionally
- ☐ Yes, frequently
- ☐ Yes, almost daily

48. Please report your height

cm

49. Please report your weight

kg
